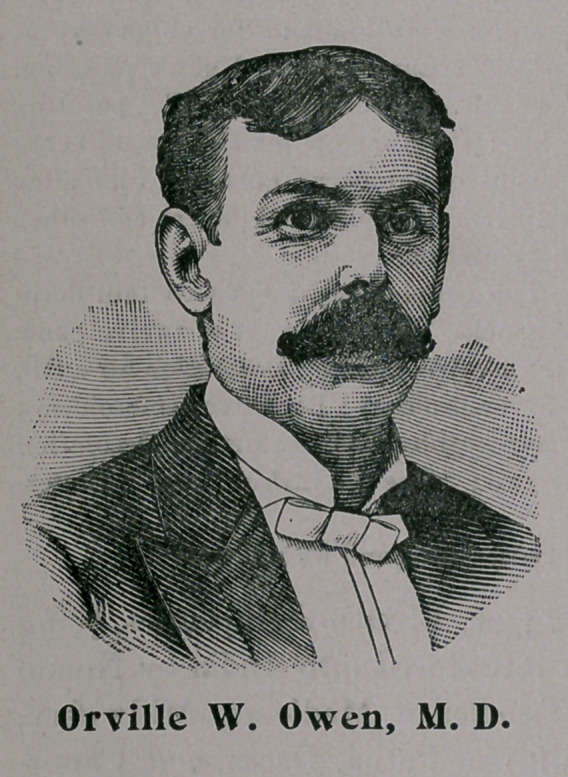# Sir Francis Bacon’s Cipher Story

**Published:** 1894-09

**Authors:** 


Sir Francis Bacon's Cipher Story. Discovered and Deciphered by Orville W. Owen, M. D. Vols. II and III. Detroit and New York: Howard Publishing Co., 1894. Paper cover, price fifty cents per volume.

The June number of The Homœopathic Physician contains a review of the first volume of the above work by an enthusiastic student and admirer of Shakespeare, Rev. E. K. Tullidge. In his review, the learned scholar ably disputes the claim that Shakespeare's plays were written by Lord Bacon. To this interesting criticism the editor of this journal can add nothing. He therefore contents himself with a brief synopsis of the three volumes, with a recommendation that all should read the volumes and make up their own opinions.

Briefly then, the author of the Cipher Story—Dr. Orville W. Owen, of Detroit, whose portrait we here give, claims to have discovered that there is in Shakespeare's plays a remarkable narrative or history of the life and times of Queen Elizabeth, cunningly concealed by being interwoven with the words of the various plays. The words of the narrative can be selected out and the narrative itself unraveled by the aid of certain words which, if known, constitute a cipher or key to the whole matter. When these words have been duly selected, set apart, and arranged in order, they constitute a description of the reign of Queen Elizabeth of the most surprising character.

**Orville W. Owen, M. D.**

The reason that this history should be so concealed is because the secrets it reveals of the Queen's private life are of such a character as would ensure the execution of the author and the destruction of his valuable works—the incomparable Shakespearean plays.

This narrative shows that Sir Francis Bacon, Viscount St. Albans, was the author of the plays usually attributed to Shakespeare. That he wrote them with the prime object of interweaving a history of Queen Elizabeth which should go down to posterity along with the plays as the products of his genius, and that he should escape detection and punishment in his own lifetime for having published court secrets.

The better to avoid detection, he paid Shakespeare, one of his companions, a large sum of money to assume the authorship of the plays.

In this story, Sir Francis Bacon informs the world that he was the son of Queen Elizabeth by the Earl of Leicester, who was privately married to her in the tower, after which he caused his previous wife, the famous Amy Robsart, to be murdered. When Francis was born, he was given over to the care of one of the ladies of the court and an associate of Elizabeth in her youth, Lady Anne Bacon, wife of Sir Christopher Bacon, to be adopted and brought up as her own son, and his identity to be kept a profound secret from himself and every one else.

By an accident of the most exciting character, which I am strongly tempted to relate, Bacon's relationship to the Queen was revealed to him by her in the presence of the court. He was so stunned by the information that he was not the son of the lady whom he called mother, and to whom he was devotedly attached, that he quitted the court and came home where he related to his foster mother the scene through which he had just passed.

Indignant at the Queen for so recklessly betraying the secret which she had herself imposed upon Lady Bacon, the latter confirmed Elizabeth's assertion, and informed Francis that his name was not Bacon but Plantagenet, and that he was the rightful heir to the throne of England. Lady Bacon then detailed the secret history of the Queen's life to the astonished Francis, and a more unsavory history of a woman in her position it would be hard to imagine.

The whole story is astounding and it is not possible for the reviewer to do justice to it. He would again recommend to the readers of this notice that they buy the book and read it for themselves.

Incredulity must, of course, rise up as a barrier to the acceptance of the story or even to the reading of it; but the debatable ground will be more clearly discerned when Dr. Owen shall have published the full key and process by which he unraveled a story so marvelous.

## Figures and Tables

**Figure f1:**